# Selection of reference miRNAs for RT-qPCR assays in endometriosis menstrual blood-derived mesenchymal stem cells

**DOI:** 10.1371/journal.pone.0306657

**Published:** 2024-07-30

**Authors:** Sabrina Yukari Santos Hacimoto, Ana Clara Lagazzi Cressoni, Lilian Eslaine Costa Mendes da Silva, Cristiana Carolina Padovan, Rui Alberto Ferriani, Júlio César Rosa-e-Silva, Juliana Meola

**Affiliations:** 1 Department of Gynecology and Obstetrics of Ribeirao Preto Medical School, University of São Paulo, Ribeirão Preto, São Paulo, Brazil; 2 National Institute of Hormones and Women’s Health (Hormona), CNPq, Porto Alegre, Rio Grande do Sul, Brazil; Northwestern University Feinberg School of Medicine, UNITED STATES

## Abstract

Choosing appropriate reference genes or internal controls to normalize RT-qPCR data is mandatory for the interexperimental reproducibility of gene expression data obtained by RT-qPCR in most studies, including those on endometriosis. Particularly for miRNAs, the choice for reference genes is challenging because of their physicochemical and biological characteristics. Moreover, the retrograde menstruation theory, mesenchymal stem cells in menstrual blood (MenSCs), and changes in post-transcriptional regulatory processes through miRNAs have gained prominence in the scientific community as important players in endometriosis. Therefore, we originally explored the stability of 10 miRNAs expressions as internal control candidates in conditions involving the two-dimensional culture of MenSCs from healthy women and patients with endometriosis. Here, we applied multiple algorithms (geNorm, NormFinder, Bestkeeper, and delta Ct) to screen reference genes and assessed the comprehensive stability classification of miRNAs using RefFinder. Pairwise variation calculated using geNorm identified three miRNAs as a sufficient number of reference genes for accurate normalization. MiR-191-5p, miR-24-3p, and miR-103a-3p were the best combination for suitable gene expression normalization. This study will benefit similar research, but is also attractive for regenerative medicine and clinics that use MenSCs, miRNA expression, and RT-qPCR.

## Introduction

Mesenchymal stem cells (MSC) are a heterogeneous subset of nonhematopoietic progenitor cells with prolonged self-renewal capacity that may differentiate in response to appropriate stimuli [1] and found in several healthy and diseased adult tissues [2], including the endometrium that is cyclically regenerated, and consequently the menstrual flow, called eMSC (endometrial MSC) and MenSCs (menstrual blood MSC), respectively [3–5]. MenSCs have gained prominence in the scientific community since their discovery in 2007 [3, 4] because of their multiple biological functions and promising applications in clinical and regenerative medicine, combined with the absence of ethical dilemmas for obtaining them [6, 7]. Their involvement have been investigated in different gynecological disorders [8] such as endometriosis [9, 10] given the importance of menstrual blood in its etiopathogenesis [11].

Although the etiopathogenic aspects of endometriosis have been widely studied, its origin remains unclear and probably results from a combination of multiple aberrant biological processes [12]. Among the various theories advocated, the implantation of endometrial tissue from the retrograde menstrual flow conducted via fallopian tubes is the most consensual theory [11]. This theory suggest that the cellular and molecular components present in menstrual blood are essential factors in endometriosis development [13, 14], such as the performance of MenSCs as ectopic lesions initiators and maintainers [9, 10, 15, 16].

Concomitant with the growing enthusiasm for studying these progenitor cells in endometriosis, vast scientific efforts have been directed towards miRNAs that may be associated with the pathophysiological process of endometriosis [17, 18]. Moreover, they are promising biomarkers for less invasive disease diagnosis [19, 20]. MicroRNAs (miRNAs) consist of small non-coding RNAs (ncRNAs) of approximately 22 nucleotides when mature, which regulate post-transcriptional gene expression through the induction of cellular machinery to degrade the target mRNA or inhibit the translation process of approximately 30% of genes [21]. These miRNAs perform essential functions in several the biological processes of the human body, maintaining cellular homeostasis [22]. Owing to their performance, they have been related to several pathological mechanisms [23], including endometriosis, in processes crucial to the establishment of the disease, such as angiogenesis [24], apoptosis [25], inflammation, and immune regulation [26]. Furthermore, the role of miRNAs in controlling the proliferation, survival, self-renewal, and differentiation of several stem cells has been discussed [27]. However, these studies have consistently reported conflicting results largely due to the methodological and analytical differences used in the different studies [28], mainly with the advent of advances in high-performance techniques, which, although present high reliability of the applied methodologies, still have validation of the findings as a critical issue [29].

Reverse transcription quantitative polymerase chain reaction (RT-qPCR) is the gold-standard technique for measuring gene expression levels, both for coding and non-coding RNAs, as it is a robust, sensitive, accurate, specific, and reproducible method for profiling expression levels, even in low-concentration samples [30]. However, the reliability of gene expression measures is affected by artifacts introduced experimentally, such as the amount of sample input, quality and integrity of the extracted material, efficiency of transcription and amplification, and particularly data normalization [31]. This, in turn, is essential for correcting non-biological variations among sample, and when performed improperly, it leads to erroneous conclusions [32]. Using multiple reference genes is the generally accepted method for normalizing RT-qPCR data [33] because the overall average miRNA expression normalization strategy, that is, the average quantification cycle (Cq) value of all miRNAs analyzed in a given experiment [34] is not a valid method when a small number of target miRNAs are evaluated [32, 35].

Reference genes, also known as internal controls, internal normalizers, and constitutive or housekeeping genes, must exhibit stable and relatively abundant expression, regardless of experimental or biological conditions [33]. However, no universal reference gene is invariably expressed under all possible conditions, and especially when referring to miRNAs, the choice of candidates is challenging because the biogenesis, size, and stability of miRNAs are different from those of mRNAs, and their biological functions are not identified completely [36]. Small nucleolar RNAs (snoRNAs), such as RNU6 (U6 small nuclear RNA), have been widely used as normalizers in RT-qPCR studies of miRNAs [35], including those on endometriosis [37–40]. Nevertheless, their robustness as reference RNAs for this type of normalization has been questioned [41], partly because they do not reflect the biochemical characteristics of miRNAs in terms of transcription, processing, and tissue-specific expression patterns [42]. Therefore, normalization of miRNAs to reference genes belonging to the same RNA class has been advocated [43, 44].

In this study, following the Minimum Information for Publication of Quantitative Real-Time PCR Experiments (MIQE) guidelines, which recommends using stable reference genes for the normalization of RT-qPCR data [45], this being the standard methodology to profile gene expression, and considering the importance of miRNAs and MenSCs for endometriosis, we evaluated the stability of 10 miRNAs (miR-30e-5p, miR-16-5p, miR-103a-3p, miR-24-3p, miR-17-5p, miR-25-3p, miR-23a-3p, miR-101-3p, miR-22-5p, and miR-191-5p) reported in the literature as more stable (**see** Table 2 **in the** Results **section**). This is the first report to suggest reference miRNAs suitable for normalizing the RT-qPCR data of target miRNAs in MSC derived from menstrual blood of patients with endometriosis. Furthermore, we present a sufficient number of reference miRNAs for studies involving these conditions. The data shown are relevant to guide the design of studies that are not only similar to ours, but also of interest to regenerative medicine and clinics that uses MenSCs.

## Materials and methods

### Ethics statement, settings, and duration

This exploratory experimental study was conducted from January to November 2022 with the approval of The Research Ethics Committee of the University Hospital of the Ribeirao Preto Medical School (HCRP 3644/2019). Samples were collected, and an *in vitro* model was established from November 09, 2014 to December 10, 2016. The collections followed the ethical guidelines established by the Declaration of Helsinki (approval number HCRP 15227/2012). All participants provided written informed consent.

We selected women from the Reference Center for Women’s Health in Ribeirao Preto (MATER) and the University Hospital Assisted Reproduction Program of Ribeirao Preto Medical School. The MenSC *in vitro* model was established at the Ribeirao Preto Hemotherapy Center of the University of Sao Paulo. We performed the study according the MIQE guidelines [45] at the Multiuser Molecular Biology Laboratory of the Department of Gynecology and Obstetrics of Ribeirao Preto Medical School of the University of Sao Paulo.

### Participants and eligibility criteria

We have previously described the clinical characteristics of the study participants [15, 46] (see Table 1 on p. 736 and p. 3, respectively). In short, the samples were collected from 18-40-year-old women with eumenorrheic menstrual cycles (interval: 24–32 ± 3 days; duration: 2–7 days) who were not on hormone therapy for at least three months before collection and with a BMI ≤30 kg/m^2^. Patients with any systemic disease, tumors, endocrinopathy, cardiovascular or rheumatological diseases, smoking, or alcohol consumption were excluded. The endometriosis group comprised women histologically and laparoscopically diagnosed with endometriosis classified as rASRM stage III (n = 6) or IV (n = 4) [47]. These patients had undergone surgical treatment for average 6 years [standard deviation (SD) ±3.7] before collection. Since stem cells have tropism for endometriotic lesions [48], the patients selected for this group had an imaging diagnosis suggestive of endometrioma during sample collection as evidence of active disease in the pelvic cavity. The healthy group comprised fertile women without a history of recurrent abortion, no clinical symptoms of endometriosis and no endometriotic lesions evidenced by laparoscopy during the tubal ligation procedure.

**Table 1:** Cq mean and standard deviation (SD) raw values for internal normalizer candidate miRNAs in relation to biological groups.

miRNA name	miRNA (ID) family	E% of RT-qPCR	Value Cq control (mean ± SD) n = 10	Value Cq endometriosis (mean ± SD) n = 10	p-value
miR-23a-3p	miR-23 (MIPF0000027)	104.4%	23.82 ± 0.97	22.97 ± 0.93	0.0821
miR-24-3p	miR-24 (MIPF0000041)	100.3%	24.00 ± 0.85	23.28 ± 1.32	0.2265
**miR-16-5p** *	miR-15 (MIPF0000006)	103.6%	25.89 ± 1.28	24.52 ± 1.42	**0.0413**
miR-25-3p	miR-25 (MIPF0000013)	96.2%	27.49 ± 1.24	26.28 ± 0.96	0.0588
miR-191-5p	miR-191 (MIPF0000194)	101.9%	28.14 ± 0.99	27.25 ± 1.35	0.2265
miR-17-5p	miR-17 (MIPF0000001)	97.8%	28.50 ± 1.55	27.41 ± 1.11	0.0696
miR-30e-5p	miR-30 (MIPF0000005)	100.9%	28.58 ± 1.28	27.21 ± 1.73	0.0696
miR-103a-3p	miR-103 (MIPF0000024)	108.7%	29.00 ± 1.10	27.95 ± 1.40	0.0963
miR-22-5p	miR-22 (MIPF0000053)	101.6%	29.13 ± 1.20	28.22 ± 1.48	0.1306

Note.

*P < 0.05.

Mann-Whitney U test (independent samples). E% = efficiency of hydrolysis probes. Family ID = identity available in the miRBase tool (https://www.mirbase.org/index.shtml, accessed on March 19, 2023) [54].

### Characterization and establishment of the *in vitro* MenSC model

We have previously established the MenSC *in vitro* model [15, 46, 49]. Menstrual blood was collected by inserting a silicone menstrual collector (Inciclo, Brazil) sterilized with gamma radiation into the vagina for 3h on the second, third, or fourth day of the menstrual cycle. We transferred the blood samples to a solution containing 1X PBS (#10010001, ThermoFisher, Waltham, MA, USA), 10X antibiotic-antimycotic solution (#15240–062, Gibco, Grand Island, NY, USA), and 10% acid citrate dextrose solution (JP Farma, São Paulo, Brazil) and stored for up to 4 h at 4°C. We then isolated the MenSCs as described earlier [3] with slight modifications [49]. Briefly, we separated the mononuclear cell layer using Ficoll-Paque density gradient centrifugation (#71-7167-00AG, GE Healthcare Bio-Sciences, Sweden) at 800xg for 30 min at 22 °C. We then transferred the interphase cells to α-MEM (Minimum Essential Medium; #11900–016, Gibco) containing 1% penicillin/streptomycin (#15140–122, Gibco), 10 mM HEPES (#H4034, Merck Millipore, Billerica, MA, USA), 20 mM sodium bicarbonate (#56297, Merck) and 15% fetal bovine serum (#SH30071.03,–HyClone; GE Healthcare Life Sciences, Logan, UT, USA). The cell culture medium was changed every 2 to 3 days until we reached 80–90% confluence for adherent cells. These cells were subcultured in 0.05% trypsin-EDTA solution (# 25300054, Gibco) until passage 3 (P3) for analysis of cell characterization (early culture) and molecular biology protocols.

According to the minimal criteria for defining multipotent mesenchymal stromal cells reported by the International Society for Cellular Therapy [50], the MenSCs are compatible with MSCs according to the immunophenotypic profile obtained for 23 markers evaluated in the FACSCalibur flow cytometer (BD Biosciences, San Jose, CA, USA) using CellQuest^™^ software version 4.0 (BD Biosciences). The results of these analyses have been presented earlier [15] (p. 736, Table 2). No significant differences were observed in the percentages of immunophenotypically labeled cells between the case and control conditions. We demonstrate the differentiation potential of these cells by inducing their differentiation into adipocytes and osteocytes as described previously [51], with modifications as described in [49]. The differentiation results can be found in [15].

**Table 2:** The literature review for the selection of candidate miRNAs for stability analysis. The miRNAs in bold were those included in the study.

Study (type of cells and tissues)	Analysis Tools	Analyzed miRNAs	Most stable	Reference
Various normal and cancerous human solid tissues.	geNorm, NormFinder.	let-7a, miR-16, miR-17-5p, miR-23a, miR-191, miR-106a, miR-103, miR-107, miR-24, miR-93, miR-25, and miR-99a.	**miR-191-5p**, miR-93, miR-106a, **miR-17–5p** and **miR-25-3p**.	[60]
Various types of human tissues.	geNorm, NormFinder, BestKeeper and ΔCt method.	Literary review, encompassing a wide variety of miRNAs.	**miR-191-5p, miR-30e-5p, miR-16-5p, miR-103a-3p** and **miR-24-3p**.	[61]
Extracellular vesicles of MCS-derived adipocytes with or without inflammation.	geNorm, NormFinder, BestKeeper and ΔCt method.	let-7a-5p, miR-16-5p, miR-23a-3p, miR-26a-5p, miR-101-3p, miR-103a-3p, miR-221-3p, miR-423-5p, miR-425-5p and miR-146a-5p.	**miR-101-3p**, **miR-23a-3p, miR-16-5p** and miR-423-5p.	[62]
Extracellular vesicles released from mesenchymal stromal cells derived from amniotic membrane.	geNorm, NormFinder, BestKeeper and ΔCt method.	let-7a-5p, miR-16-5p, miR-22-5p, miR-23a-3p, miR-26a-5p, miR-29a-5p, miR- 101-3p, miR-103a-3p, miR-221-3p, miR-423-5p, miR-425-5p, miR-660-5p and RNU6.	**miR-101-3p** and **miR-22-5p**.	[63]
Plasma from women with surgically confirmed endometriosis and disease-free control women	2ΔCq; Mann-Whitney Test.	miR-148b-3p, miR-30e-5p and miR-103a-3p.	miR-148b-3p and **miR-30e-5p**.	[64]
Bone marrow mesenchymal stromal cells (BM-MSCs), HS5, and HS27A stromal cell lines in normoxia and hypoxia conditions	geNorm; ReFinder	miR-16-5p, miR-34b-3p, miR-103a-3p, miR-191-5p, let-7a-5p, and RNU6A	**miR-191-5p** and **miR-16-5p**	[65]
Extracellular vesicles of cartilage, adipose tissue, and bone marrow cells	geNorm, NormFinder, BestKeeper and ΔCt	let-7a-5p, miR-16-5p, miR-22-5p, miR-23a-3p, miR-26a-5p, miR-29a-5p, miR- 101-3p, miR-103a-3p, miR-221-3p, miR-423-5p, miR-425-5p and miR-660-5p	**miR-103a-3p** and **miR-22-5p**	[66]
Extracellular vesicles of bone marrow-derived mesenchymal stromal cells in osteoarthritis model	geNorm, NormFinder, BestKeeper and ΔCt; RefFinder	let-7a-5p, miR-16-5p, miR-22-5p, miR-23a-3p, miR-24-3p, miR-26a-5p, miR-29a-5p, miR-34a-5p, miR-101-3p, miR-103a-3p, miR-221-3p, miR-423-5p, miR-425-5p, miR-660-5p, and U6 snRNA	**miR-24-3p** and **miR-16-5p**	[67]

### Isolation, total RNA integrity, and cDNA synthesis of mature miRNAs

Total RNA was extracted from all samples during the same period using the Allprep DNA/RNA/miRNA Universal Kit (#80224, Qiagen, Valencia, CA, USA) according to the manufacturer’s instructions. The samples were treated with Ambion DNA-free DNase Treatment and Removal (#AM1906, Invitrogen, Carlsbad, CA, USA) to remove gDNA contamination. Total RNA integrity was determined using an Agilent RNA 6000 Nano Kit (#5067–1511, Agilent, Palo Alto, CA, USA) on an Agilent 2100 Bioanalyzer (Agilent Technologies). Samples with RNA integrity number (RIN) ≥ eight were included in the study. Subsequently, the total RNA concentrations of the intact samples were measured using the Qubit^®^ 2.0 fluorometer (ThermoFisher) using the Qubit RNA BR Assay Kit (#Q10210, Invitrogen). A concentration of 100ng total RNA was used for miRNA cDNA synthesis using the TaqMan Advanced miRNA cDNA synthesis kit (#A28007, ThermoFisher) according to the manufacturer’s instructions. The kit uses a 3’ poly-A tailing and 5’ ligation of an adapter sequence to extend the mature miRNAs on each end before reverse transcription. The mature miRNAs were reverse-transcribed to cDNA by recognizing universal sequences at the 5’ and 3’ ends using universal RT primers. The cDNA was 1:10 diluted and pre-amplified using Universal miR-Amp Primers and miR-Amp Master Mix to uniformly increase the cDNA amount for each target while maintaining the relative differential expression levels. The final product (cDNA–pre-amp) was stored at −20°C for use in qPCR.

### RT-qPCR and amplification efficiency test

RT-qPCR was performed with the TaqMan Advanced miRNA assay (# A25576, Applied Biosystems) for the 10 reference candidate miRNAs: hsa-miR-30e-5p (479235_mir), hsa-miR-16-5p (477860_mir), hsa-miR-103a-3p (478253_mir), hsa-miR-24-3p (477992_mir), hsa-miR-17-5p (478447_mir), hsa-miR-25-3p (477994_mir), hsa-miR-23a-3p (478532_mir), hsa-miR-101-3p (477863_mir), hsa-miR-22-5p (477987_mir), and hsa-miR-191-5p (477952_mir). Each amplification reaction was prepared in triplicate, in a 96-well plate, with a 10 μL volume containing 5.0 μL TaqMan Fast Advanced Master Mix (2X) (# 4444557, Applied Biosystems), 0.5 μL hydrolysis probe (20X), 2.5μL cDNA–pre-amp (diluted 1:10) and 2.0μL nuclease-free water. We considered technical replicates with up to 0.3 cycles difference between Cq values. PCR runs were performed on the ViiA Real-Time PCR System equipment (Applied Biosystem) under the following conditions: one cycle at 50 °C for 2 min, one cycle at 95 °C for 20 s, 40 cycles of 1 s at 95 °C and 20 s at 60 °C. A negative control (nuclease-free water instead of template) was included in all plates and showed no amplification (Cq = “undetectable”). This study evaluated the efficiency (E%) of 10 hydrolysis probes using a standard curve with serial dilutions of 1:2 constructed using seven points from a pool of equal amounts of cDNA–pre-amp from all samples. The equipment software was used to calculate E%. A 1:10 dilution was selected for the study, and E% between 90 and 110% was accepted as the recommendation [52].

### Differential expression and co-regulation of reference candidate miRNAs

To check the NormFinder requirements [53], we applied the Mann–Whitney U test for independent samples after transforming raw Cq to 2 ^ Cq to detect the differences in candidate miRNA expression between the case and control groups. These analyses were performed using MedCalc Statistical Software version 20 (MedCalc Software Ltd, Ostend, Belgium; https://www.medcalc.org; 2020), and the significance level was defined as P value < 0.05. The miRbase database release 22.1 (https://www.mirbase.org/index.shtml, accessed on March 19, 2023) [54] was also searched to check whether the selected miRNAs did not belong to the same gene family, thus respecting the requirements of the geNorm [33] and comparative Ct [55] statistical algorithms.

### Evaluation of the stability of expression of candidate miRNAs as reference

Initially, we performed an exploratory analysis using descriptive statistics to profile the expression variation of the 10 candidate miRNAs for the reference gene. Subsequently, we used the RefFinder web-based tool (available at https://www.heartcure.com.au/reffinder/, accessed on January 15, 2023) [56] to classify the miRNAs based on their stability values obtained by the BestKeeper [57], Cycle comparative threshold (ΔCt method) [55], geNorm [33], and NormFinder [53] algorithms using the mean Cq values from the three technical replicates for each sample. RefFinder provides a general ranking based on the stability classification for each algorithm individually, allocates an appropriate weight to each miRNA, and calculates the geometric mean of their weights to obtain the final classification [56].

Additionally, we used NormFinder (Excel add-in) version 0.953 (https://moma.dk/normfinder-software) to compare the classification of miRNAs with the NormFinder (RefFinder) algorithm since this algorithm in RefFinder does not consider intergroup variations [49, 58]. Furthermore, we used geNorm (qbase plus) version 3.2 [59], considering the E% values shown in Table 1. geNorm calculates the stability value (*M*) for each candidate based on the average pairwise variation of each target. We performed this analysis to compare the results obtained from geNorm (qbase plus) using the calculated E% with the geNorm (qbase plus) ranking, assuming a PCR efficiency of 100% for calculating *M* [49, 58]. In addition, using geNorm (qBase plus), we calculated the normalization factor (NF) which determines the minimum number of miRNAs required for reliable normalization. Calculations started with the two most stable miRNAs (lowest M values), and geNorm continually added another miRNA and recalculated the NF. The ideal number of reference genes is reached when the pair variation value (VNF) is below the recommended cutoff value (0.15) [33].

## Results

**Fig 1:**
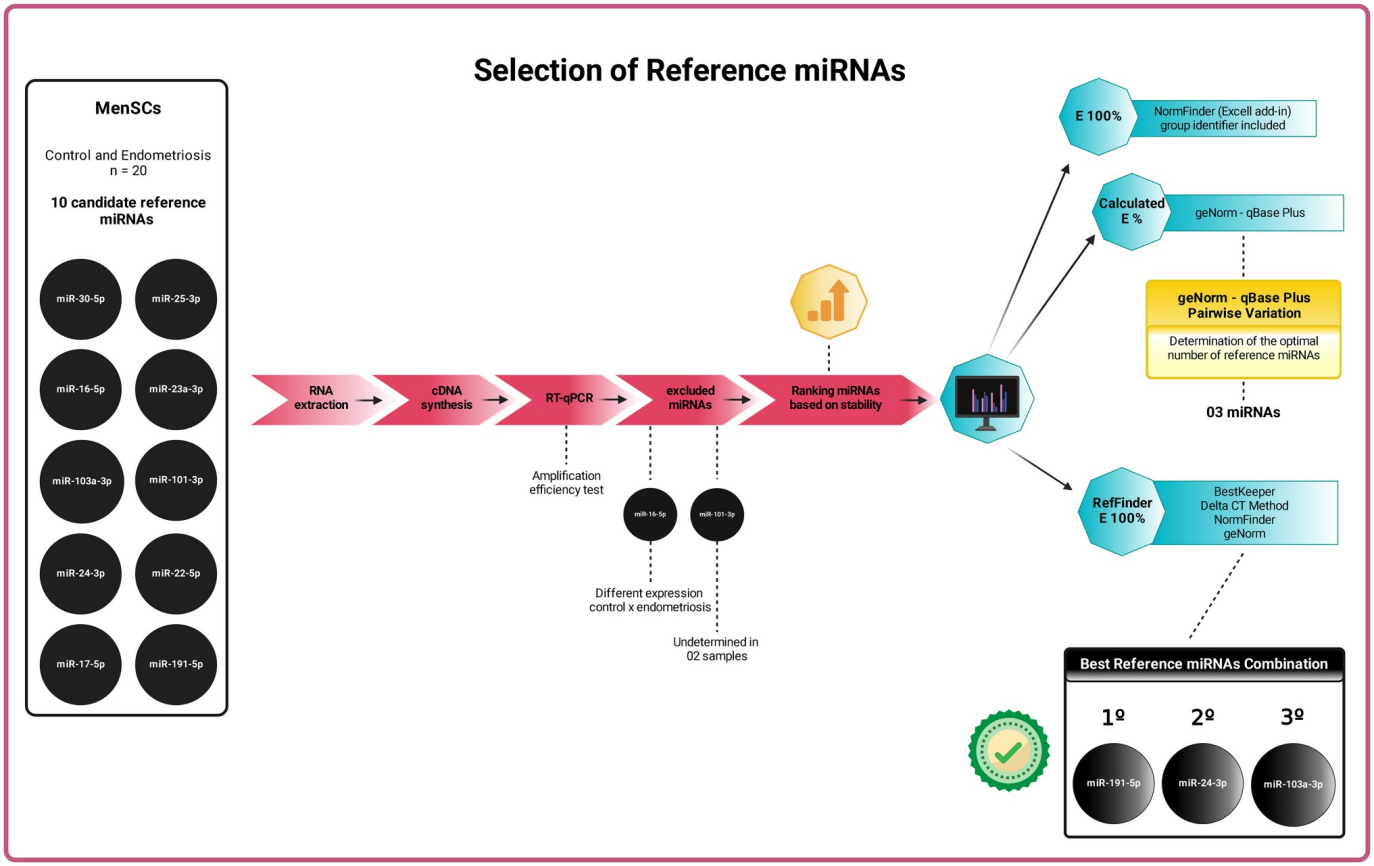
Infographic of study results. To select candidate miRNAs as a reference in two-dimensional cultures of MenSCs from women with and without endometriosis, we followed the strategy: 1) establishment of the MenSC model in vitro; 2) selection of 10 stable candidate miRNAs in MSCs, endometriosis, and in different healthy and tumor tissues; 3) evaluation of expression by RT-qPCR and exclusion of inappropriate miRNAs; 4) analysis of raw Cq data by geNorm (qBase plus), NormFinder (excel add-in) and RefFinder (four algorithms implemented); 5) final stability classification by RefFinder; 6) identification of three reference miRNAs sufficient for adequate normalization of RT-qPCR data; and 7) selection of the best combination: miR-191-5p, miR-24-3p and miR-103a-3p. Created with BioRender.com (2024).

This exploratory study evaluated the stability of 10 miRNAs known as reference genes for RT-qPCR under experimental conditions involving MSCs, endometriosis, and different healthy and tumor tissues, in addition to being commercially recommended (Table 2). To this end, we explored their expression under experimental conditions involving the two-dimensional cultures of MenSCs from 10 healthy women and 10 women with endometriosis (Fig 1).

The clinical data have been described previously [15, 46] (p. 736; p. 3, respectively). We have not observed significant differences for age (mean 36±3.0 and 35±3.5 year), body mass index (mean 25.18±2.9 and 26.03±2.4), and menstrual flow collection days (mean 2.7±0.8 and 2.7±0.6 d) among the case and control groups. The patients had advanced endometriosis (stage III or IV), pelvic pain, and infertility.

### Expression profile of candidate miRNAs for internal normalizers

**Fig 2:**
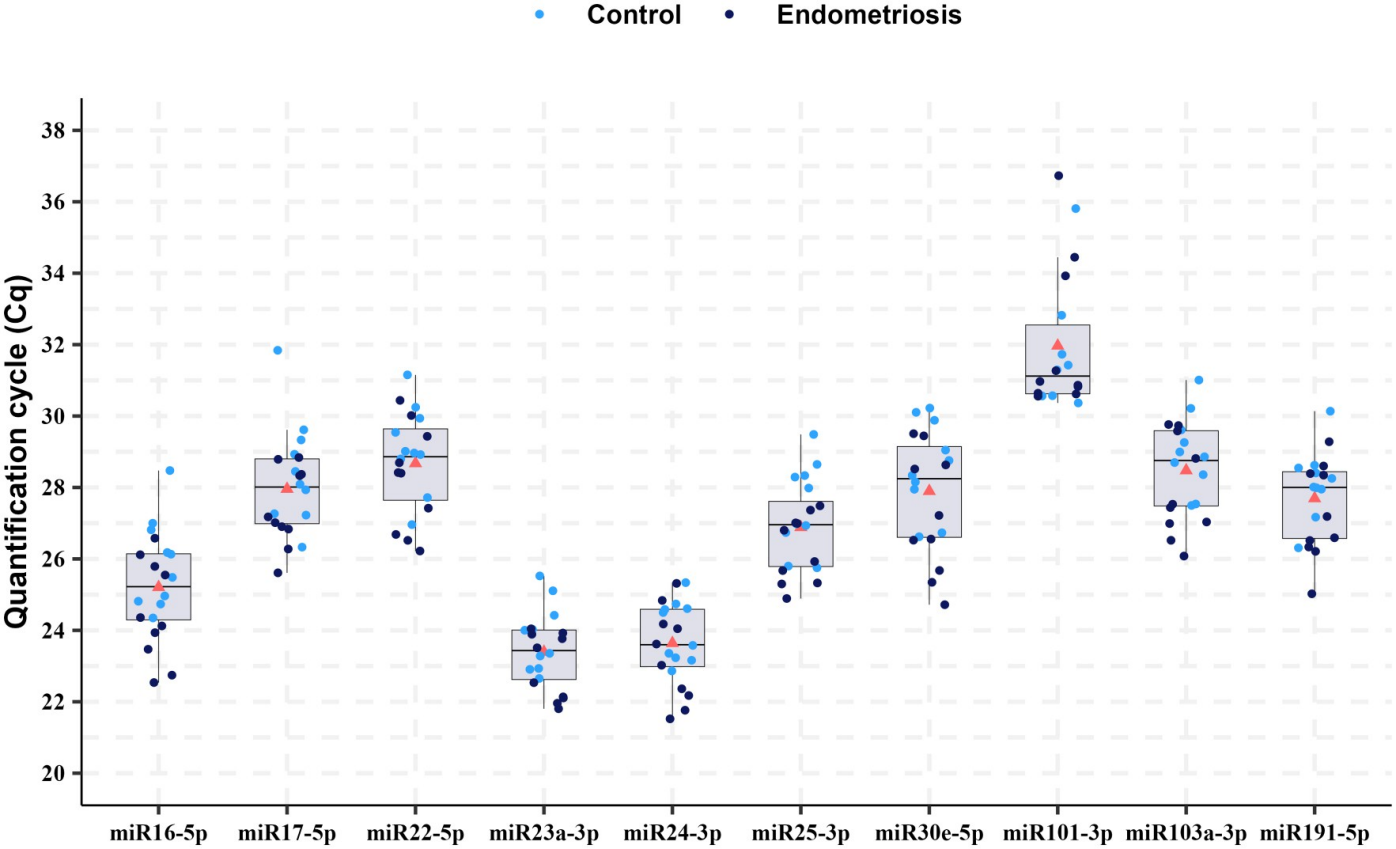
Overview of Cq values obtained by RT-qPCR for all samples. The points represent the mean raw Cq values for technical replicates of individual samples. The boxes correspond to the interquartile range (IQR) for each miRNA. The lines within the boxes indicate the median values of Cq and the red triangle the mean value. The minimum and maximum values are respectively [Q1 − (1.5*IQR)] and [Q3 + (1.5*IQR)]. This boxplot was generated using the ggplot2 package in RStudio (v3.5.1, accessed on May 23, 2024) [68].).

The variability in the raw values of Cq or Ct (quantification or threshold cycle) of the samples is shown for all miRNA candidates as internal control normalizers in Fig 2 (S1 Table). Among the evaluated miRNAs, the Cq of approximately 70% candidates were identified between 24 and 30 cycles, with the most abundantly expressed being miR-23a-3p and miR-24-3p (Cq min around 21 and max 25) and the least expressed being miR-101-3p (Cq min of 30.3). Furthermore, miR-101-3p was excluded from the stability analyses because it was not detected in all samples.

The mean raw Cq values and SD between the biological groups are shown in Table 1; all Cq values lower in the endometriosis group than that in the healthy group. However, there were no significant differences compared with that in the control group, except for miR-16-5p (P = 0.04). This gene was excluded from the stability analyses because the NormFinder algorithm requires the average expression levels between the evaluated conditions to be similar [53]. Thus, miR-30e-5p, miR-103a-3p, miR-24-3p, miR-17-5p, miR-25-3p, miR-23a-3p, miR-22-5p, and miR-191-5p were used for stability analyses.

### MiRNA family, RT-qPCR efficiency, and internal normalizing miRNAs ideal for RT-qPCR

**Fig 3:**
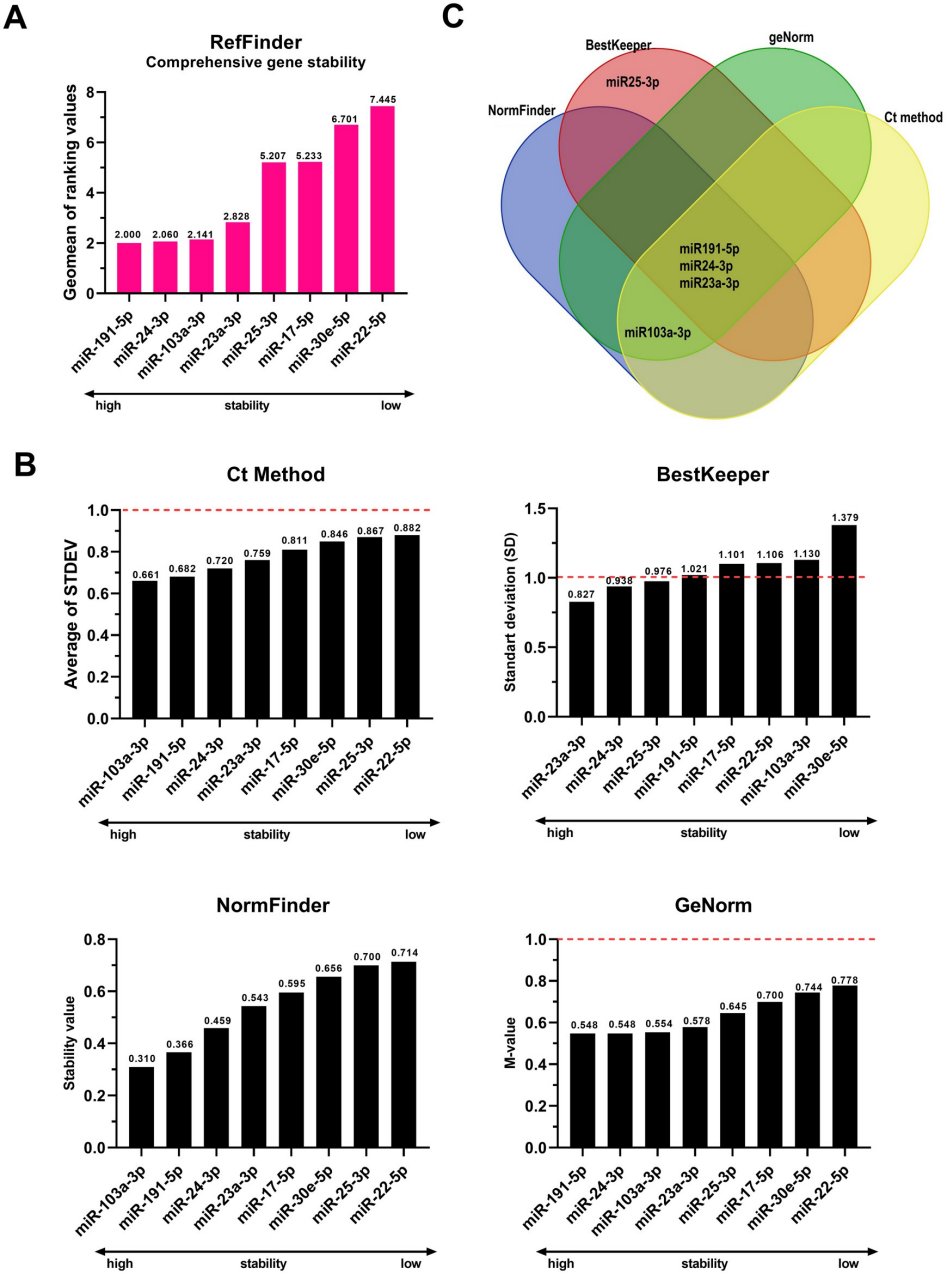
Comprehensive stability ranking performed by RefFinder (A), and individual rankings generated by each algorithm separately for candidate miRNAs as internal normalizers (B). Overlap between the four most stable candidate miRNAs indicated by each of the algorithms applied for the stability test (C). The dashed lines show the cutoff value as recommended by BestKeeper [57], comparative Ct (ΔCt method) [55], and geNorm [33]. Graphs constructed using GraphPad Prism version 8.0.1 (GraphPad Software, San Diego, Califórnia, EUA. www.graphpad.com, accessed on 19 March 2023). Web tool available for Venn diagram (http://bioinformatics.psb.ugent.be/webtools/Venn/, accessed on March 19, 2023).

Coregulated genes may increase the chances of being falsely classified as stably expressed by methods that use the pairwise comparison approach [69]; therefore, we checked that the eight candidate miRNAs for internal normalizers did not belong to the same family according to the miRbase database release 22.1 (https://www.mirbase.org/index.shtml, accessed on March 19, 2023) [54] (Table 1). The comprehensive stability rating and stability score generated by RefFinder [56], as well as the individual ratings for each algorithm (Ct method [55], BestKeeper [57], NormFinder [53], and geNorm [33]) are shown in Fig 3A and 3B, respectively. The overlap of the four most stable candidate miRNAs indicated by each of the algorithms tested is presented in the Venn diagram in Fig 3C, constructed using a tool available on the web (http://bioinformatics.psb.ugent.be/webtools/Venn/, accessed on March 19, 2023). The four most stable miRNAs in the general ranking (RefFinder) were miR191-5p > miR-24-3p (present in all four algorithms) > miR-103a-3p (present in geNorm, NormFinder, and comparative Ct) > miR-23a-3p (present in all algorithms).

Because the geNorm and NormFinder algorithms available in RefFinder have significant limitations [58], we evaluated the stability of the candidates using NormFinder (Excel add-in, available at https://moma.dk/normfinder-software, accessed on March 19, 2023) and geNorm (qBase plus) [59].

**Fig 4:**
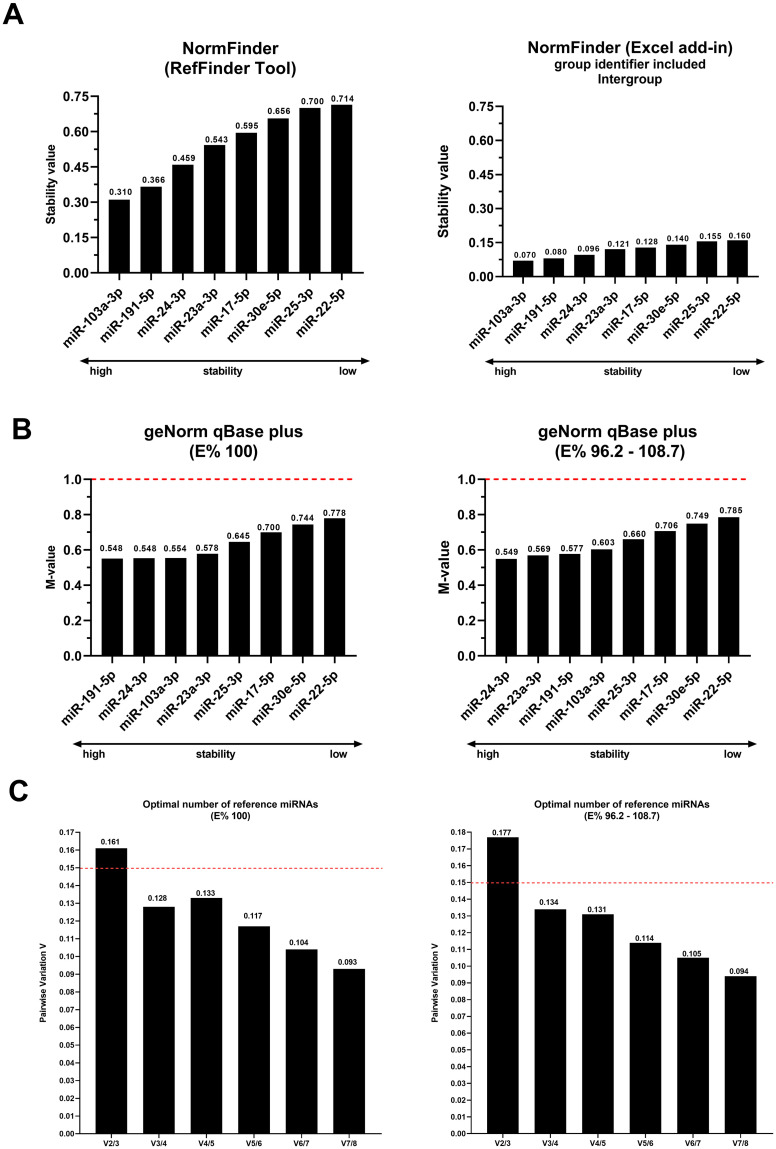
The ranking was based on the stability of reference candidate miRNAs according to the NormFinder (RefFinder) and NormFinder (Excel add-in, including intergroup evaluation) (A). Classification considering the percentages of 100% efficiency and the calculated efficiencies (E% ranging from 96 to 108%) of geNorm (qBase plus) (B). Pairwise variations obtained by geNorm (qBase plus) considering the percentage of E% 100 and the percentage of calculated E% (C). The dashed lines show the cutoff value as recommended by BestKeeper [57], comparative Ct (ΔCt method) [55], and geNorm [33]. Graphs constructed using GraphPad Prism version 8.0.1 (GraphPad Software, San Diego, California, USA. www.graphpad.com, accessed on March 19, 2023).

NormFinder consists of a mathematical model that estimates the general variation intra- and intergroup gene expression, combining the results with a single stability value [53]. When the group identifier was included in the analysis, the absolute stability measures in the NormFinder (Excel add-in) calculation differed from those in NormFinder (RefFinder) (Fig 4A). However, the classification of the genes remained the same; therefore, the combined inter- and intragroup analyses is unlikely to be relevant for identifying the most stable reference miRNAs for the sample set tested.

geNorm classifies genes based on their stability value, with the *M* of good reference genes being <0.5 under homogeneous conditions and between 0.5 and 1 when evaluating a heterogeneous set of samples (in our case, cultured cells from sick and healthy participants) [69]. Thus, all the miRNAs analyzed were suitable for this mathematical model, as the values of *M* were between 0.5 and 0.7. When percentages of efficiency were included in the analysis, the absolute stability measures in the calculation of geNorm (qBase plus) differed from those of geNorm (RefFinder). Interestingly, the ranking order changed among only the four most stable candidates (Fig 4B). However, despite the ranking changes, variations in E% (96–108%) were unlikely to affect the final normalization result, as internal normalizing miRNAs were the same. Using geNorm (qBase plus), we determined the optimal number of reference normalizing miRNAs using the recommended cutoff value of 0.15 for paired variation between normalization factors (VNF). We applied this analysis to two conditions: using all E% = 100% and considering the calculated E% (Table 1). The first VNF value < 0.15 was detected at V3/4 in both analyses, suggesting that three stable miRNAs (miR-191-5p, miR-24-3p, and miR-103a-3p) were required for reliable normalization (Fig 4C).

## Discussion

To the best of our knowledge, no study has systematically evaluated the stability of candidate miRNAs as reference genes in MenSCs from healthy women or those with endometriosis for application in studies profiling target miRNAs using RT-qPCR. Selecting appropriate internal controls is critical for normalizing and, consequently, ensuring the interexperimental reproducibility of data obtained using the gold-standard technique for expression quantification [70]. As miRNAs fine-tune gene expression at the post-transcriptional level and play integral roles in virtually all physiological processes, influencing health and disease outcomes [71], identifying miRNAs with stable expression presents a significant challenge due to their unique physicochemical and biological characteristics [35, 36]. Here, we highlight that miR-191-5p, miR-24-3p, and miR-103a-3p are more stable for normalizing experimental conditions involving two-dimensional cultures of endometriosis MenSCs.

Variations in the Cq values of reference genes are crucial when selecting a normalization gene. We noted that the Cq of 70% analyzed miRNAs were within the appropriate detection range (24 to 30 cycles). Ideally, the expression levels of reference genes should not be exceedingly low (Cq > 32) or high (Cq < 15) [72] and closely resemble those of the target genes studied [73]. Moreover, all the examined miRNAs demonstrate a trend of upregulated expression in endometriosis, as evidenced by lower Cq values than those in healthy women. This observation aligns with the recognized pathological role of miRNAs in endometriosis, where they act as key regulators of cellular processes such as proliferation, invasion, epithelial-mesenchymal transition (EMT), and angiogenesis [74].

However, caution must be exercised when interpreting statistical presentations based on raw Cq values, because they are non-linear [75]. Hence, we employed multiple algorithmic approaches, including geNorm [33], NormFinder [53], BestKeeper [57], and comparative Ct [55], which utilize distinct approaches to enhance the reliability of the findings. Notably, except for BestKeeper, the statistical models classified the same four miRNAs in distinct positions as highly stable (Fig 3B). To consolidate the rankings in our study, we used RefFinder, a widely adopted web-based tool [56] that integrates these mathematical models simultaneously [76]. It is worth noting that this integrative resource has important limitations regarding the geNorm and NormFinder algorithms implemented in the tool [49, 58] as it does not estimate intergroup variations via NormFinder and overlooks amplification efficiencies via geNorm. Nevertheless, when the RefFinder results were compared with geNorm (qBase plus) and NormFinder (Excel add-in) results, these gaps were unlikely relevant to our study since the four most stable miRNAs were always the same.

ncRNAs are typically categorized into two main groups based on their functions. Housekeeping ncRNAs, such as small nuclear RNAs (snRNAs) and small nucleolar RNAs (snoRNAs), regulate fundamental cellular processes, whereas regulatory ncRNAs, such as microRNAs (miRNAs), small interfering RNAs (siRNAs), and long non-coding RNAs (lncRNAs), regulate gene expression at various levels, including epigenetic, transcriptional, and post-transcriptional [77]. For example, miRNAs can independently regulate a broad spectrum of genes. Owing to their inherent regulatory properties, they are implicated in normal physiological and pathological conditions [71], such as endometriosis [17–20]. Consequently, the search for abundantly and ubiquitously expressed miRNAs poses significant challenges. Thus, another class of non-coding RNAs are commonly used as normalizers for miRNA expression.

The snoRNAs RNU6A and RNU6B are among the most frequently employed reference genes in miRNA studies [35], including endometriosis investigations [40, 78–80]. However, their application may be inappropriate [33, 41] because snoRNAs such as RNU6 fail to accurately represent the physicochemical properties of miRNA molecules concerning transcription, processing, production, degradation, and tissue-specific expression patterns [42]. For these reasons, their use as reference genes may introduce bias in quantifying target miRNA expression [35, 43, 44, 81]. Thus, in light of these considerations, this study opted not to analyze this class of RNAs.

After an extensive review of miRNAs with proven expression stability under experimental conditions similar to those in this study, we identified the most stable miRNAs: miR-101-3p, miR-23a-3p, miR-16-5p, miR-103a-3p, miR-24-3p, and miR-22-5p in MSCs [62, 63, 66, 67]; miR-30e-5p in endometriosis [64]; and miR-191-5p, miR-17-5p, miR-25-3p, miR-103a-3p and miR-24-3p in both normal and tumor tissue conditions [60, 61]. Considering the comprehensive ranking provided by RefFinder, cost-effectiveness, and our results suggesting the use of minimum three genes for optimal normalization, we recommend using miR-191-5p, miR-24-3p, and miR-103a-3p as internal controls for this study design.

MiR-191-5p is a member of the miR-191/425 cluster located within the first intron of the *DALRD3* gene on chromosomal region 3p21.31 [54]. This cluster is highly conserved across various metazoan species, suggesting its important involvement in higher eukaryotes [82]. MiR-191 is implicated in numerous diseases, including oncogenesis, as well as several physiological processes such as apoptosis, proliferation, cell cycle, development, and differentiation [83]. Despite its involvement in anomalous conditions, miR-191 is a suitable reference gene for bone marrow mesenchymal stromal cells [65] and certain solid tumors [60]. MiR-24-3p is part of the miR-23b/27b/24 cluster located within an intron of the *C9orf3* gene in the chromosomal region 9q22.32 [54]. This miRNA plays a pivotal role in regulating the cell cycle and numerous cancer hallmarks, including apoptosis, proliferation, metastasis, invasion, angiogenesis, autophagy, and drug resistance [84]. Notably, miR-24-3p overexpression mitigates endometrial inflammation and modulates endometritis pathogenesis [85]. Nevertheless, some studies have reported its stable expression across normal tissues and healthy sera [86, 87]. miR-103a-3p is located in chromosomal region 5q34 and belongs to the miRs-103/107 family, which plays a crucial role in regulating various characteristics of epithelial stem cells, thereby maintaining stem cell niche integrity and facilitating self-renewal [88]. Its dysregulation has been linked to chronic pain [89] and oncogenic functions in breast cancer [90]. However, miR-103a-3p has been consistently stable in solid tumors [60] and extracellular vesicles derived from cartilage, adipose tissue, and bone marrow cells [66].

The strength of our study is that we applied multiple widely accepted algorithms to determine suitable reference miRNAs in samples with stringent selection criteria. Furthermore, we evaluated a panel with 10 miRNAs as being more stable under experimental conditions close to ours. Despite its advantages, this study had some limitations. The main limitation of our study is the need for more validation for the selected reference miRNAs, as we did not perform RT-qPCR analysis of a specific target miRNA. Identifying target miRNAs as biomarkers to distinguish between MenSCs with endometriosis and those without this condition remains a significant challenge. We believe adequate validation will only be possible with a larger sample size and when more target miRNAs become accessible in the literature. Despite meeting the requirements outlined in the statistical algorithms employed, the sample size is limited. In addition, the expression profile after culture must be interpreted with caution as it does not always represent *in vivo* systems, that is, the natural cellular microenvironment. However, *in vitro* studies remain the most appropriate method for research in this field.

## Conclusions

We advocate using three miRNAs, miR-191-5p, miR-24-3p, and miR-103a-3p, as internal controls for normalizing RT-qPCR data in studies with similar experimental designs, considering both accuracy and cost-effectiveness. Our findings identified miR-191-5p, miR-24-3p, and miR-103a-3p as the most stable genes, whereas miR-30e-5p and miR-22-5p were unstable. This breakthrough can leverage research on endometriosis stem cells and miRNAs by enhancing the interexperimental reproducibility of gene expression data obtained using RT-qPCR. Furthermore, it may positively affect regenerative medicine and clinical applications that utilize MenSCs, miRNA expression profiling, and RT-qPCR analysis.

## Supporting information

S1 Table Raw Cq data obtained by RT-qPCR for the control and endometriosis groups. Undetermined–sample without PCR amplification.
(DOCX)
