# Supplementary material for: Selection of reference miRNAs for RT-qPCR assays in endometriosis menstrual blood-derived mesenchymal stem cells
Source: PLoS One. 2024 Jul 30;19(7):e0306657. doi: 10.1371/journal.pone.0306657 (PMC11288454; doi:10.1371/journal.pone.0306657)
Supplement: S1 Table — Undetermined–sample without PCR amplification. (DOCX) [file pone.0306657.s001.docx]

| S1 Table: Raw Cqs data obtained by RT-qPCR for the control and endometriosis groups. | | | | | | | |  |  |  |  |
| --- | --- | --- | --- | --- | --- | --- | --- | --- | --- | --- | --- |
|  |  |  |  |  |  |  |  |  |  |  |  |
| Sample Name | Group | miR-30e-5p | miR-25-3p | miR-24-3p | mir-23a-3p | miR-22-5p | miR-191-5p | miR-17-5p | miR-103a-3p | miR-101-3p | miR-16-5p |
| C10 | control | 26.732 | 26.739 | 23.579 | 23.355 | 27.717 | 27.952 | 27.265 | 27.537 | 30.367 | 24.815 |
| C17 | control | 26.621 | 25.752 | 22.863 | 22.907 | 26.961 | 26.31 | 26.329 | 27.497 | 30.563 | 24.347 |
| C22 | control | 28.338 | 25.798 | 23.356 | 22.652 | 29.938 | 27.169 | 27.934 | 28.698 | 35.81 | 25.483 |
| C29 | control | 30.223 | 28.646 | 25.336 | 25.523 | 31.153 | 30.136 | 31.841 | 31.008 | 31.73 | 26.999 |
| C31 | control | 27.95 | 26.98 | 23.162 | 23.282 | 29.014 | 28.012 | 27.226 | 28.359 | 30.569 | 24.962 |
| C32 | control | 29.047 | 29.486 | 24.499 | 25.108 | 28.8 | 28.546 | 28.091 | 30.215 | 31.426 | 28.474 |
| C34 | control | 28.756 | 27.984 | 23.233 | 22.933 | 29.543 | 28.407 | 28.449 | 28.86 | 31.291 | 24.736 |
| C35 | control | 29.882 | 28.291 | 24.738 | 24.421 | 30.246 | 28.622 | 29.331 | 28.995 | undetermined | 26.815 |
| C38 | control | 28.154 | 26.935 | 24.608 | 24.002 | 28.965 | 28.254 | 28.93 | 29.259 | 32.823 | 26.179 |
| C39 | control | 30.105 | 28.334 | 24.584 | 24.028 | 28.922 | 27.992 | 29.615 | 29.613 | undetermined | 26.132 |
| E2 | endometriosis | 25.345 | 25.927 | 21.763 | 22.107 | 26.685 | 26.211 | 27.177 | 26.521 | 30.556 | 22.745 |
| E3 | endometriosis | 28.518 | 27.487 | 24.046 | 23.924 | 30.439 | 28.345 | 28.368 | 29.733 | 36.73 | 25.79 |
| E4 | endometriosis | 24.718 | 24.892 | 21.522 | 22.137 | 26.222 | 25.023 | 25.61 | 26.08 | 30.618 | 22.538 |
| E7 | endometriosis | 29.506 | 26.802 | 24.837 | 23.891 | 30.016 | 28.601 | 28.789 | 29.763 | 34.447 | 26.117 |
| E8 | endometriosis | 29.445 | 26.992 | 24.177 | 23.767 | 28.693 | 28.39 | 28.839 | 28.813 | 30.639 | 25.549 |
| E9 | endometriosis | 26.525 | 27.009 | 23.023 | 23.511 | 27.421 | 27.187 | 27.015 | 27.438 | 30.826 | 24.358 |
| E11 | endometriosis | 28.632 | 27.367 | 25.315 | 24.043 | 29.432 | 29.281 | 28.332 | 29.582 | 33.926 | 26.58 |
| E12 | endometriosis | 25.676 | 25.299 | 22.364 | 21.958 | 26.52 | 26.334 | 26.277 | 27.034 | 31.27 | 23.47 |
| E13 | endometriosis | 27.218 | 25.327 | 23.616 | 22.535 | 28.4 | 26.515 | 26.903 | 27.531 | 30.864 | 24.124 |
| E27 | endometriosis | 26.556 | 25.674 | 22.175 | 21.805 | 28.418 | 26.592 | 26.836 | 26.99 | 30.969 | 23.935 |
| Note: undetermined - sample without PCR amplification | | | | |  |  |  |  |  |  |  |
